# Surveillance of panicle positions by unmanned aerial vehicle to reveal morphological features of rice

**DOI:** 10.1371/journal.pone.0224386

**Published:** 2019-10-31

**Authors:** Daisuke Ogawa, Toshihiro Sakamoto, Hiroshi Tsunematsu, Toshio Yamamoto, Noriko Kanno, Yasunori Nonoue, Jun-ichi Yonemaru

**Affiliations:** 1 Institute of Crop Science, National Agricultural and Food Research Organization, Tsukuba, Japan; 2 Institute for Agro-Environmental Sciences, National Agriculture and Food Research Organization, Tsukuba, Japan; Clemson University, UNITED STATES

## Abstract

Rice plant architecture affects biomass and grain yield. Thus, it is important to select rice genotypes with ideal plant architecture. High-throughput phenotyping by use of an unmanned aerial vehicle (UAV) allows all lines in a field to be observed in less time than with traditional procedures. However, discrimination of plants in dense plantings is difficult, especially during the reproductive stage, because leaves and panicles overlap. Here, we developed an original method that relies on using UAV to identify panicle positions for dissecting plant architecture and to distinguish rice lines by detecting red flags attached to panicle bases. The plant architecture of recombinant inbred lines derived from Japanese cultivars ‘Hokuriku 193’ and ‘Mizuhochikara’, which differ in plant architecture, was assessed using a commercial camera-UAV system. Orthomosaics were made from UAV digital images. The center of plants was plotted on the image during the vegetative stage. The horizontal distance from the center to the red flag during the reproductive stage was used as the panicle position (PP). The red flags enabled us to recognize the positions of the panicles at a rate of 92%. The PP phenotype was related to but was not identical with the phenotypes of the panicle base angle, leaf sheath angle, and score of spreading habit. These results indicate that PP on orthomosaics could be used as an index of plant architecture under field conditions.

## Introduction

Phenotyping is important for the evaluation and selection of crop lines in breeding. Researchers evaluate various traits such as leaf water status, leaf color (chlorophyll content), the number of culms, plant size and architecture, of each line in the field using the traditional procedures, which are often labor- and time-consuming. On the other hand, recently developed high-throughput phenotyping technologies have begun to be used for monitoring plant growth, disease occurrence and damage under abiotic stresses [1–4]. One of these technologies relies on the use of unmanned aerial vehicles (UAVs), which are convenient and powerful tools for phenotyping of field crops[5]. By using aerial images obtained from UAVs, researchers can evaluate the crop lines in larger experimental field with less time and labor-work than the traditional procedures. UAVs have a strong point to observe the whole plot rather than an individual leaf and plant of lines, allowing to overcome individual phenotypic variability. Some UAVs are commercially available with affordable price. These lead to time- and cost-saving for phenotyping. Moreover, UAVs have an ability to take hundreds of images a flight and can mount different types of cameras or sensors to take red-green-blue (RGB), multispectral, hyperspectral, and thermal images, which can provide information on plant density, height, water status, leaf temperature, and disease severity[1–10]. In particular, RGB images can be relatively easily analyzed by user-friendly software such as Pix4D Mapper[11] and Metashape (developed from PhotoScan)[6, 8]. Data from UAVs can be useful for the genetic analysis of traits[7, 9, 10] and estimation of yield[12–15]. Therefore, high-throughput phenotyping using UAVs may innovate breeding methods.

Plant architecture influences biomass and grain yield in crops; one of the best examples of this came with the introduction of the semi-dwarf phenotype in the Green Revolution [16–19]. But the same plant architecture generally has both advantages and disadvantages for crop cultivation[20, 21]. Plants with a spreading habit cover furrows, inhibiting weed growth through shading, but they cover a large area, limiting the number of plants that can be grown in a field. In rice, there is a risk that panicles contact the wet ground, reducing grain quality because of preharvest sprouting. Conversely, plants with a compact habit and erect leaves can be grown densely, increasing yield [22] possibly by increasing light capture ability especially at high latitude. However, compact habit may increase the risk of competition with weeds and neighboring plants. When these risks and benefits are balanced, the most appropriate plant types should differ among crops and production regions. Although plant architecture is genetically controlled [7, 10, 16–23], plants often adapt it to field conditions [24–26]. Therefore, plant architecture should be investigated in production fields.

Panicle position (PP) is one of the determinants of plant architecture and may be an important trait for determining the optimal distance between plants in a rice cultivation field and for preventing panicles from touching the wet ground. To make breeding more efficient, PP could be evaluated by UAV, but individual plants are indistinguishable, especially during the reproductive stage, in densely planted rice fields. To identify the PP of individual rice plants in UAV images, we attached red flags to the panicle bases, identified the flags in an orthomosaic derived from UAV images, and calculated PP as a measure of plant architecture. To reveal what PP relates to as an indicator, we compared it with other traits including leaf sheath angle (LSA), panicle base angle (PBA), and score of spreading habit (SSH) evaluated by eye in the field.

## Materials and methods

### Cultivation of RILs and parental cultivars

The members of the Institute of Crop Science, the National Agricultural Research Organization (NARO) previously developed recombinant inbred lines (RILs) from ‘Hokuriku 193’ (HO) and ‘Mizuhochikara’ (MI) by single seed descent and named them HOMI-RILs [27]. We examined 81 HOMI-RILs (F_8_) and the parental cultivars for this study.

Seeds soaked in water at 28°C for 2 days were sown in trays filled with soil on 16 April 2018 and incubated at 30°C in the dark for 2 days. Seedlings were grown in a paddy field in the Kannondai District in Tsukuba (Japan) and were covered with thin plastic film to protect them from the cold. On 16 May, we transferred 33 seedlings per line (11 plants 18 cm apart × 3 rows 30 cm apart, no replicates) to another paddy field in the Kannondai District and grew them according to standard procedures at the NARO in Tsukuba.

**Fig 1:**
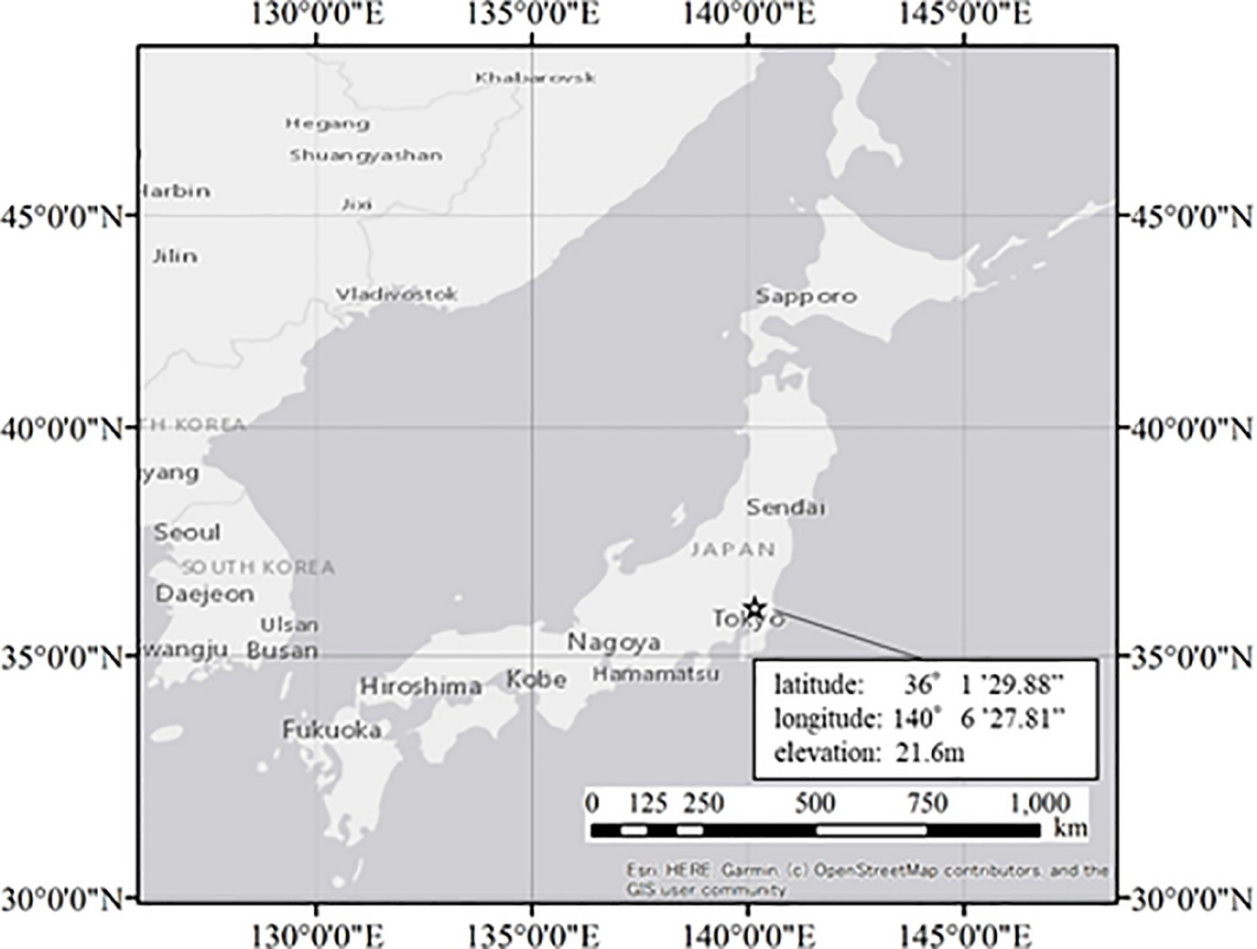
Location of our paddy field, the Kannondai District in Tsukuba (Japan). Location of the Kannondai District marked with a star.

Location of the Kannondai District is shown in Fig 1. The map was made with ArcGIS software (ESRI Japan, Tokyo, Japan). The meteorological data (S1 Table) were obtained from the Weather Data Acquisition System of the Institute for Agro-Environmental Sciences, NARO[28].

### Field observation by unmanned aerial vehicle

Phantom 4 Pro (P4P; DJI, Shenzhen, China), a small UAV released in 2016, was used for taking aerial photographs (S1 Fig). The P4P’s digital camera has a 1-inch 20-megapixel complementary metal-oxide semiconductor (CMOS) sensor (valid pixels only), and provides an 84-degree field of view. The UAV automatically flew above the field where the 81 HOMI-RILs and parental cultivars were grown. The flight pattern and image capture were controlled by DJI GS Pro software on a tablet computer (iPad mini 4, Apple, Cupertino, CA, USA). Two aerial surveys of 9 min each were conducted from 8 to 9 am: on 18 June (235 images; vegetative stage, before heading) and 20 September (238 images; reproductive stage, after heading).

The DJI GS Pro software and the camera used the following settings: capture mode, time interval; speed, 1.0 m/s; altitude, 10.3 m; front overlap ratio, 80%; side overlap ratio, 79%; gimbal pitch angle, −90°, image size, 3:2 (5472 × 3648 pixels); image format, JPG; white balance, cloudy; aperture, auto; shutter, auto; exposure compensation value, −1. To set the focus, the P4P was manually raised to 10 m, the camera was focused automatically on a region of the canopy on the iPad, and then the focus mode was changed to manual.

Before the first aerial survey, we painted black and white markers at 8 points on paved surfaces surrounding the field as ground control points (GCPs), and precisely measured the latitude, longitude, and altitude of each point with a TCRP1205 surveyor (Leica, Heerbrugg, Switzerland).

### Mapping and calculation of PP in an orthomosaic derived from aerial images

We used Agisoft PhotoScan Professional v. 1.4.3 software (Agisoft, St. Petersburg, Russia) to generate an orthomosaic from each set of aerial images in the following steps: (1) align photos (accuracy, high), (2) input GCPs, (3) build mesh (surface type, height field; source data, sparse cloud), (4) build digital elevation model (DEM; source data, sparse cloud), (5) calibrate colors (source data, model; calibrate white balance, no), (6) build orthomosaic (surface DEM; blending mode, mosaic). The orthomosaics used in this study are shown in S2 and S3 Figs.

The orthomosaics were imported into ENVI v. 5.5 remote sensing software (Harris Geospatial, Boulder, CO, USA). The map projection was converted to UTM zone 54N (WGS-84) with a 2-mm/pixel resolution. The converted image was rotated by 66° clockwise to match the long-side direction of the test field with the lateral direction of the final output image. Then the image was cropped to a rectangle (28 000 × 14 000 pixels) including the field and the 8 markers to minimize the file size and thus processing time.

**Fig 2:**
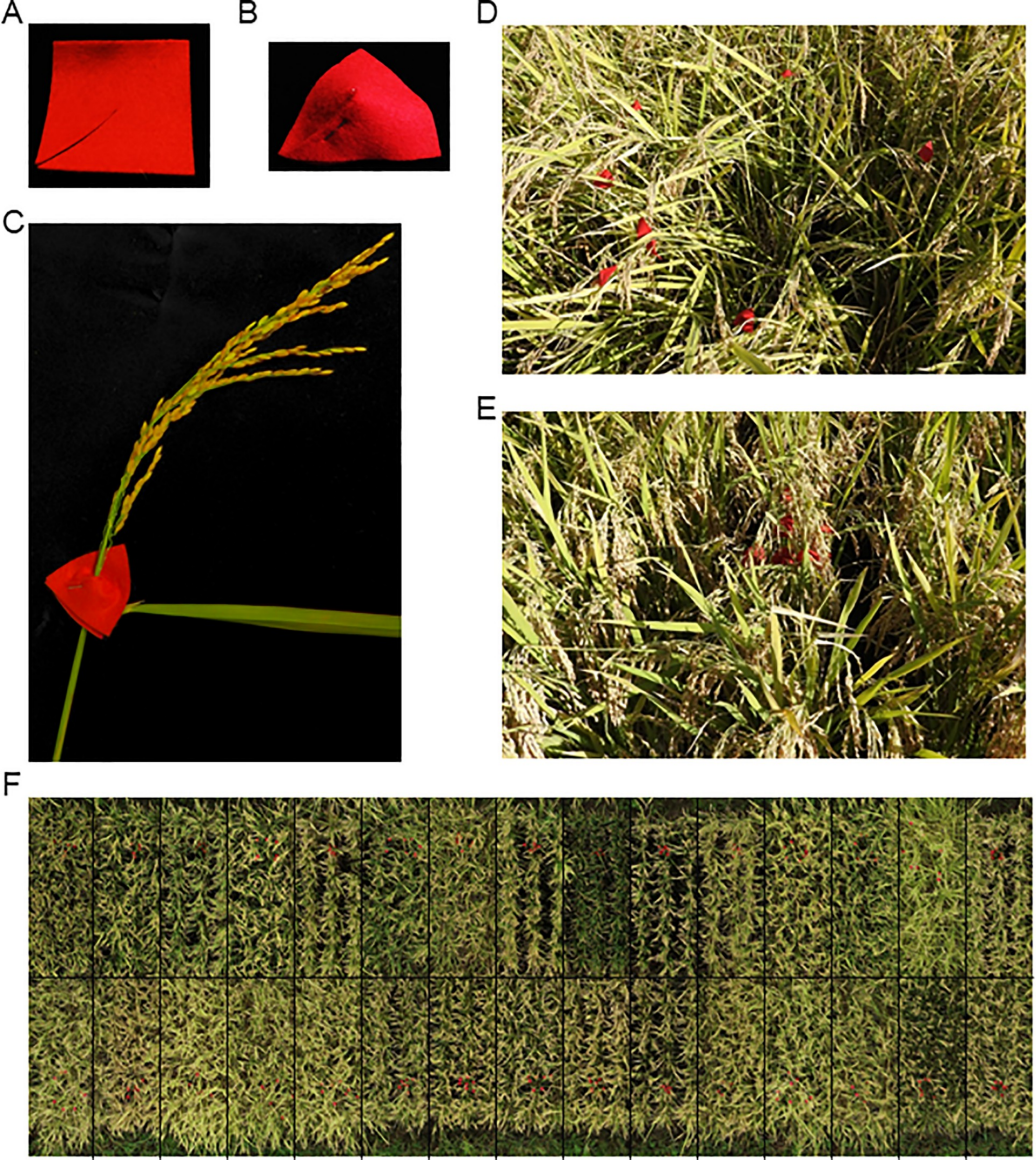
Red flags attached to panicle bases. (A) A square of red felt (4.5 cm × 4.5 cm) was cut from one corner to the center by scissors and (B) stapled around (C) a panicle base. (D, E) The spread of the red flags on the panicles depended on the HOMI-RIL (photographed from above). (F) The red flags were attached to every third plant in the middle row. This orthomosaic was made from photographs taken on 20 September 2018. The black lines delineate the HOMI-RILs.

**Fig 3:**
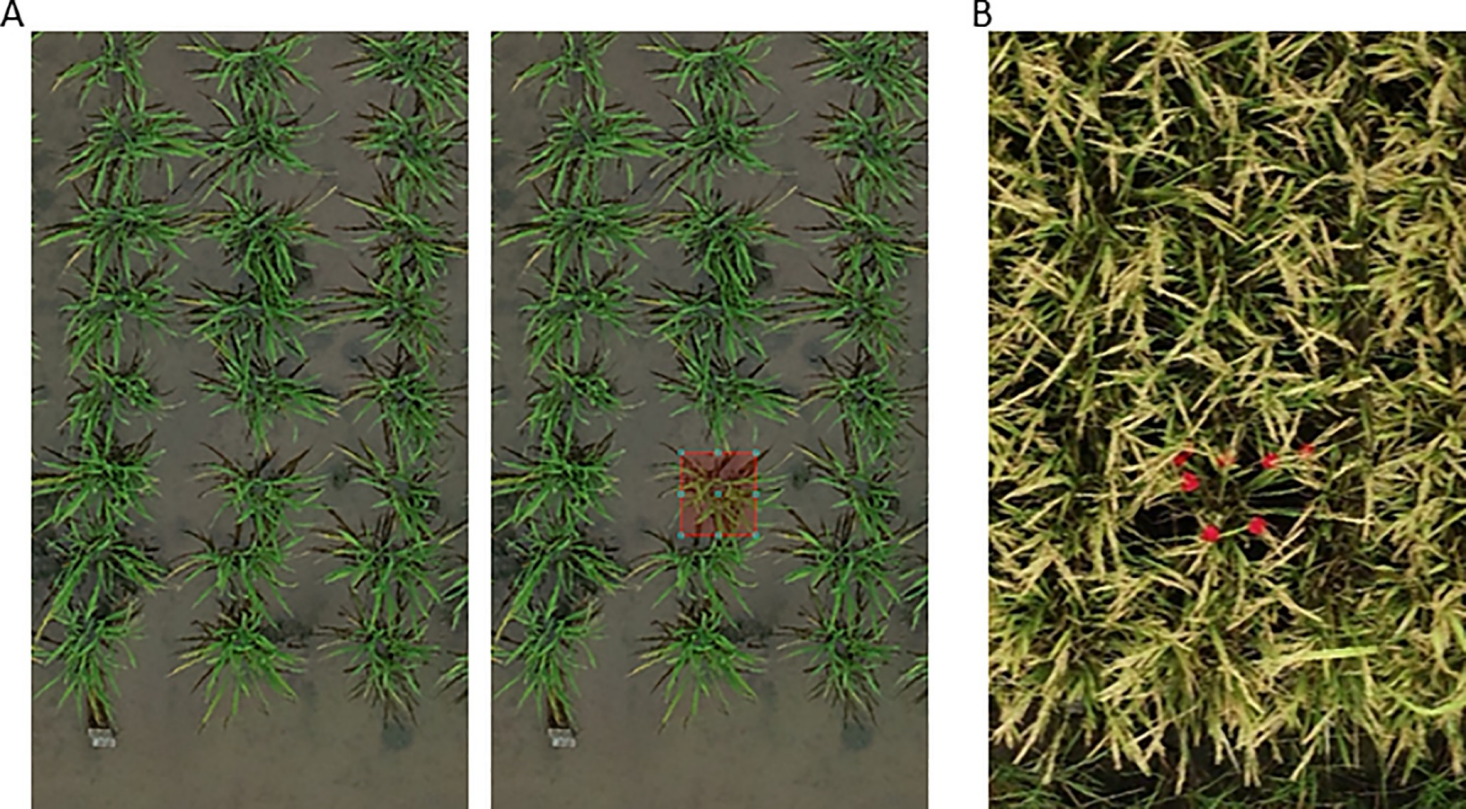
**Detection of plant centers in orthomosaics at (A) vegetative stage and (B) reproductive stage.** (A) The images were viewed in ENVI software and the position of the centers selected by eye were recorded using the software (left, before; right, after selection). (B) The positions of the red flags were selected by eye in ENVI software, and the average distance from the center to the red flags was calculated as PP.

To identify the positions of panicles in individual plants, we attached flags to the panicle bases. By comparing colors, materials, and sizes (S2 Table), we chose 4.5-cm × 4.5-cm red felt flags (Fig 2A–2E). If a plant had more than 8 panicles, we fixed the red flags to 8 panicles; otherwise, we fixed the flags to all panicles. We fixed the flags at the panicle base of every third plant in the middle row of a plot to show PP (Fig 2F) on 18 September, and took images using the UAV on 20 September. The center of plants during the vegetative stage and the position of the red flags during the reproductive stage were plotted by eye in ENVI 5.5 (Fig 3). Average horizontal distance between paired points was used as PP.

### Assessment of plant spreading habit at maturity

PBA and LSA were measured with a laser range finder (GLM 50 C, Bosch, Gerlingen, Germany) in the field. SSH was evaluated by eye in the field on a scale of 1 (compact) to 5 (spreading), before the red flags were attached.

### Principal component analysis

Data of LSA, PBA, SSH, and PP in HOMI-RILs were used for principal component analysis (PCA) of principal components (PCs) 1 to 4 in R version 3.3.3 software (https://www.r-project.org/) for quantitative trait locus (QTL) analysis.

### Measurement of yield traits

For measurement of panicle weight (PW, g) and stem + leaf weight (SLW, g), shoots of mature plants were dried for over a month in a drying room and cut 3 cm below the panicle base to separate the parts. For measurement of fertilized-grain weight (GW, g) per plant, all grains were removed from panicles, and awns, rachis branches, and unfertilized grains were removed using a PS-100 system with a vacuum cleaner (PSS, Ishinomaki, Japan). Grain number (GN) was counted with a Waver IC-VA counter (Aidex, Nagoya, Japan). The 1000-grain weight (TGW, g) was based on 500 grains. Days to heading (DTH) was calculated from sowing to the day when 6 of the 11 plants in the middle row had headed. Culm length (CL, cm) from ground to panicle base, panicle length (PL, cm) from panicle base to panicle tip of the longest culm, and panicle number (PN) were recorded in the field from 2 weeks to a month after heading. Panicle weight per panicle (PWPP, g), grain weight per panicle (GWPP, g), and grain number per panicle (GNPP) were calculated as PW, GW, and GN divided by PN.

### QTL analysis

Genotype data of 163 indel markers (S1 File) and phenotype data of HOMI-RILs were used for QTL analysis. Following PCR with a KAPA2G Fast PCR kit (Roche, Basel, Switzerland) and DNA electrophoresis in 3% agarose gel, we constructed genetic maps in MAPMAKER/EXP v. 3.0b software[29]. QTL analysis was performed by composite interval mapping in QTL Cartographer v. 2.5 software[27], and the threshold was calculated from 1000 permutations.

### Sequencing of *TAC1*

The sequences of *Tiller Angle Control 1* (*TAC1*) alleles in HO and MI were determined from short-read Illumina resequencing data [27, 30]. The sequences are shown in S2 File.

## Results and discussion

### Characteristics of HOMI-RILs

**Fig 4:**
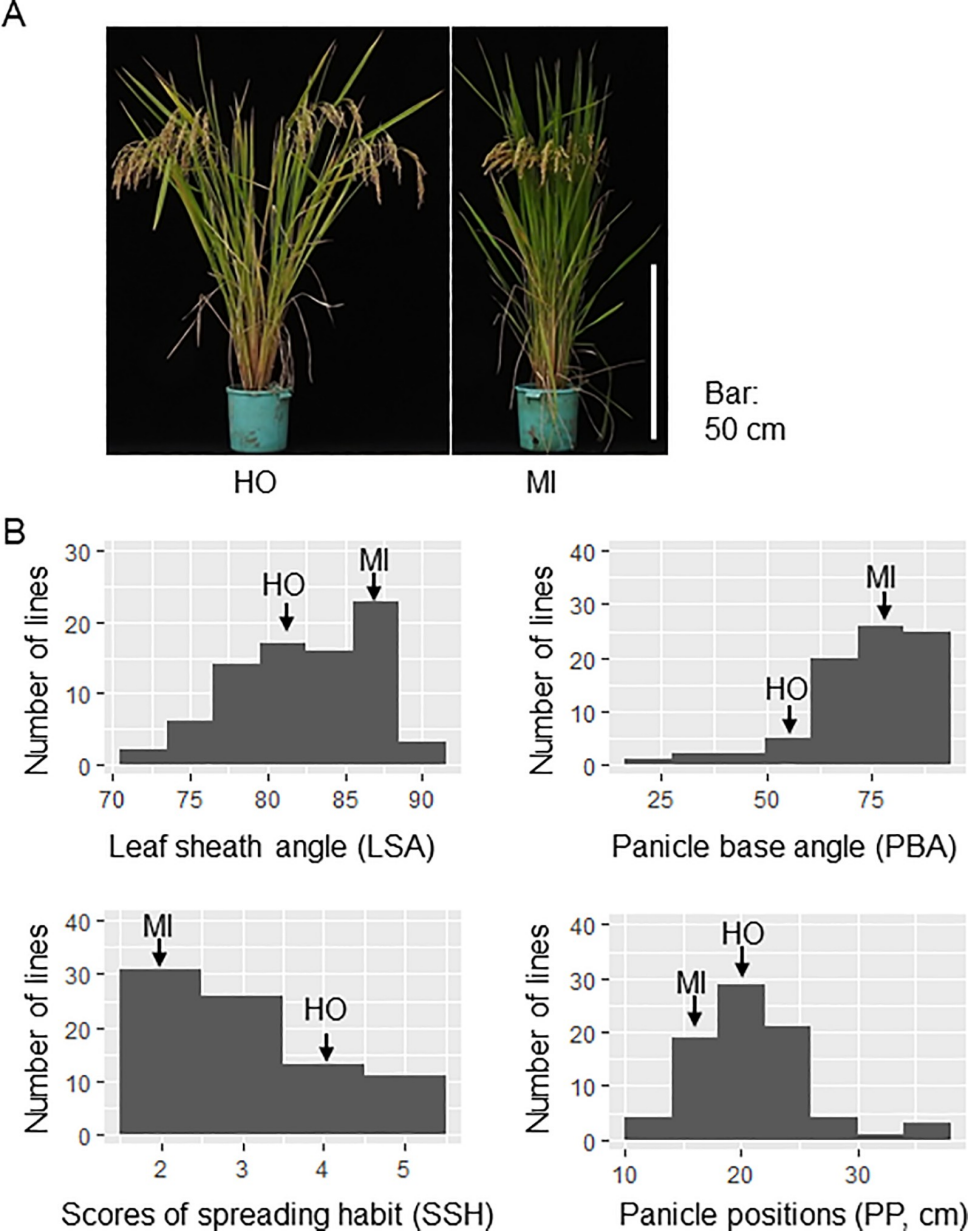
Plant spreading habit of HOMI-RILs. (A) ‘Hokuriku 193’ (HO) and ‘Mizuhochikara’ (MI) at reproductive stage. (B) Histograms of LSA, PBA, SSH and PP. The width of the values corresponding to each bar is 3 (LSA), 11 (PBA), 1 (SSH) and 4 (PP). Arrows indicate parental values.

‘Hokuriku 193’ (HO) and ‘Mizuhochikara’ (MI) are high-yielding semi-dwarf cultivars grown in Japan that differ in plant architecture. HO has a spreading habit with inclined culms and leaves, and MI has a compact habit with vertical culms and leaves (Fig 4A). Resequencing analysis identified HO as *indica* and MI as *japonica* [27]. HO had a lower PN than MI but a longer PL and CL, which may be responsible for its higher GN and GW (S4 Fig). The LSA and PBA were significantly lower in HO than in MI (S5 Fig). The difference in the plant architecture motivated us to develop and evaluate HOMI-RILs derived from them. The phenotypic distributions of LSA, PBA, SSH, and PP in the HOMI-RILs segregated beyond those of the parents (Fig 4B).

### PP detected in orthomosaic as a new index of plant architecture

We characterized PP because this index is obtained from our original method using the UAV systems and may have a potential to understand plant architecture in the field. We examined how often the red flags are found in the orthomosaic of the 81 HOMI-RILs and parents during the reproductive stage. As a result, 92% (591/643) of the red flags were detected. We could mark the position of all the center of plants in the orthomosaic during the vegetative stage. As described in the Material and method, we calculated horizontal distance between the red flags and the center of the plants for obtaining values of PP. These results indicated that our method can provide data with a high probability.

**Fig 5:**
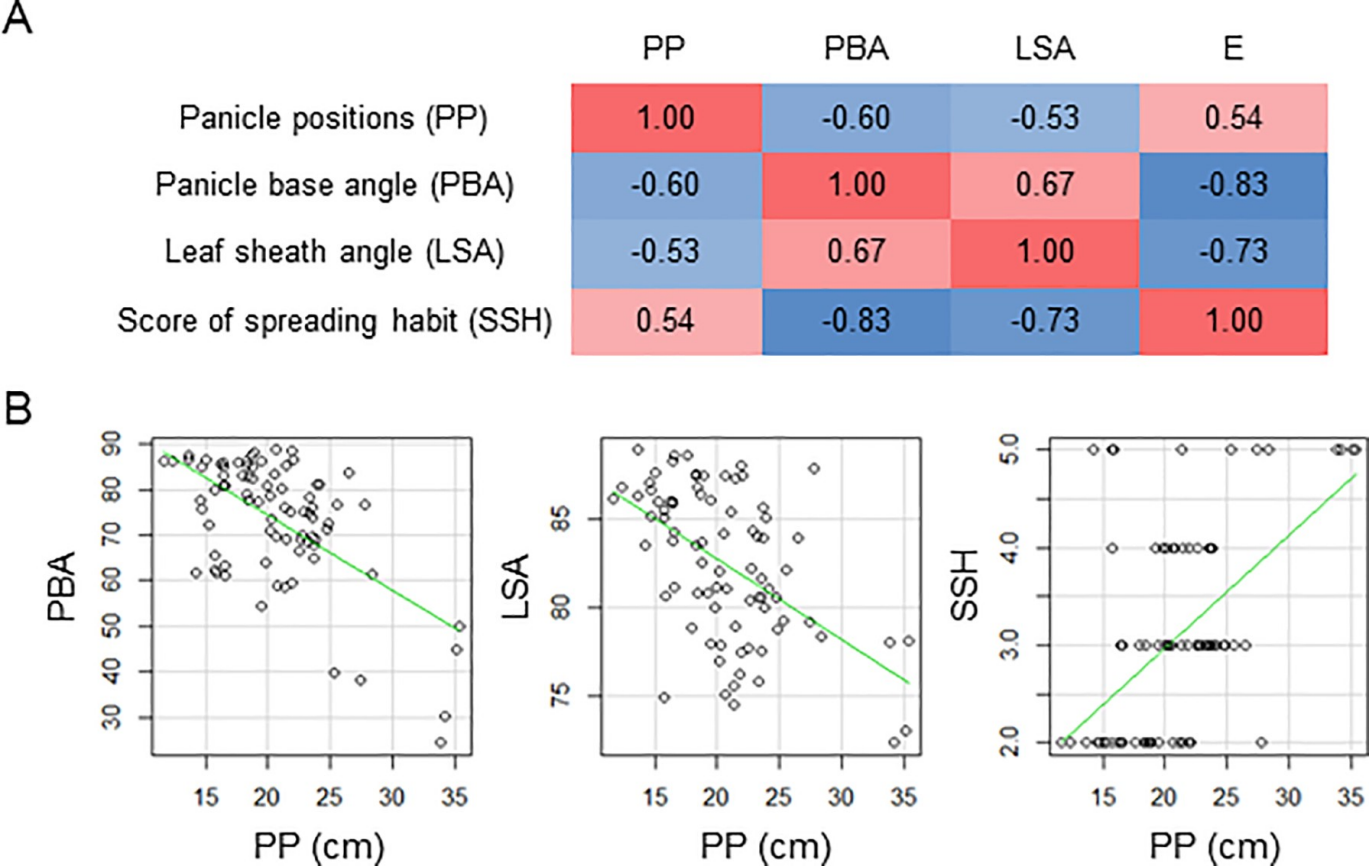
Relationships between PP and related data. (A) Pearson correlation coefficients. All correlations were significant (*P* < 0.001). (B) Scatter plots of PP against PBA, LSA, and SSH. Fitted lines are green.

To understand what this index is like, we characterized PP as a phenotype related to spreading habit. We first compared it with LSA, PBA, and SSH in the HOMI-RILs (Fig 5). The correlation between PBA and SSH was the greatest (*R* = −0.83), implying that the researcher who scored SSH paid more attention to PBA than to LSA. The correlations between the three indices and PP were not as strong, although they were all significant (P<0.001); that between PP and PBA was the greatest (*R* = −0.60). This result may be expected because both PP and PBA are related to panicle, whereas LSA is measured on leaf sheaths hidden from above. These results indicate that PP is an index similar to LSA, PBA, and SSH, but measures a different aspect of plant architecture.

Next, we characterized the genetics of PP by performing QTL analysis with the data on PP, LSA, PBA, and SSH in the HOMI-RILs (Table 1) and PCs obtained from PCA on the data (S3 Table). QTL analysis revealed several loci for the indices (Table 1). QTLs with the highest LOD for PP (*qPP9*), LSA (*qLSA9*), PBA (*qPBA9-1*, *2*), SSH (*qSSH9*), and PC1 (*qPC1_9*) were detected at Indel_544 (20.5 Mb) or Indel_577 (22.9 Mb) on chromosome (Chr.) 9. This commonality supports the similarity of PP to the other indices. *Tiller Angle Control 1* (*TAC1*, Os09t0529300), at 20.7 Mb on Chr. 9, regulates rice tiller angle [31]. Analysis of the *TAC1* sequence in HO and MI (S2 File) revealed that the MI allele is the same as the *japonica* Nipponbare allele, which produces compact plants, and the HO allele is a typical *indica* allele, leading to a spreading habit. This result indicates that *TAC1* is a candidate gene for *qPP9*, *qLSA9*, *qPBA9-1*, *2*, *qSSH9*, or *qPC1_9*. In contrast, the other QTLs for plant architecture did not show commonality with the indices. These QTLs may be responsible for the differences among PP, LSA, PBA, SSH, and PCs as indices of plant architecture.

**Table 1:** QTL analysis of traits related to spreading habit (in bold) and yield in HOMI-RILs.

Trait	Name of QTLs	Chr.	Closest markers	Mb^a^	LOD	AE(%)^b^	R^2^ ^c^	Candidate genes or QTLs
Days to heading	*qDTH1-1*	1	Indel_10	3.2	5.5	2.42	0.18	-
Days to heading	*qDTH1-2*	1	Indel_14	5.7	4.2	2.96	0.25	-
Grain number per panicle	*qGNPP2*	2	Indel_92	2.4	3.3	7.56	0.12	-
Panicle length	*qPL2*	2	Indel_139	23.8	4.6	0.95	0.17	-
**PC3 of PCA**	***qPC3_3***	**3**	**Indel_155**	**4.2**	**3.0**	**0.24**	**0.16**	**-**
Panicle length	*qPL3*	3	Indel_165	9.3	3.3	-0.73	0.10	-
Grain number per panicle	*qGNPP3*	3	Indel_762	25.3	3.7	8.01	0.14	-
Panicle weight	*qPW4*	4	Indel_271	30.5	3.5	-3.13	0.13	-
Panicle number	*qPN4-1*	4	Indel_271	30.5	7.4	-1.49	0.26	*NAL1*
Panicle number	*qPN4-2*	4	Indel_276	33.4	5.1	-1.48	0.25	*NAL1*
Stem and leaf weight	*qSLW5-1*	5	Indel_322	22.6	7.2	-5.87	0.32	-
Stem and leaf weight	*qSLW5-2*	5	Indel_324	23.7	7.0	-5.94	0.34	-
**Scores of spreading habit**	***qSSH6-1***	**6**	**Indel_386**	**24.5**	**4.3**	**-0.47**	**0.19**	**-**
**Scores of spreading habit**	***qSSH6-2***	**6**	**Indel_805**	**26.0**	**4.3**	**-0.33**	**0.09**	**-**
**PC1 of PCA**	***qPC1_6***	**6**	**Indel_805**	**26.0**	**3.5**	**0.44**	**0.06**	**-**
Culm length	*qCL6*	6	Indel_805	26.0	10.0	-5.39	0.34	*qIN3-6*
Panicle length	*qPL6*	6	Indel_392	28.0	3.6	-0.82	0.12	*qPL-6*
Culm length	*qCL7*	7	Indel_437	18.8	3.5	-3.07	0.10	-
**Panicle position**	***qPP9***	**9**	**Indel_544**	**20.5**	**6.3**	**-2.55**	**0.26**	***TAC1***
**Panicle base angle**	***qPBA9-1***	**9**	**Indel_544**	**20.5**	**3.3**	**5.32**	**0.12**	***TAC1***
**Leaf sheath angle**	***qLSA9***	**9**	**Indel_544**	**20.5**	**14.2**	**3.13**	**0.49**	***TAC1***
**Scores of spreading habit**	***qSSH9***	**9**	**Indel_544**	**20.5**	**14.6**	**-0.70**	**0.39**	***TAC1***
**PC1 of PCA**	***qPC1_9***	**9**	**Indel_544**	**20.5**	**14.8**	**1.09**	**0.38**	***TAC1***
**Panicle base angle**	***qPBA9-2***	**9**	**Indel_547**	**22.9**	**6.5**	**7.19**	**0.25**	***TAC1***
Days to heading	*qDTH10*	10	Indel_584	16.9	3.6	2.09	0.11	*Ehd1*
Thousands grain weight	*qTGW10*	10	Indel_586	17.3	5.2	-1.01	0.20	-
Panicle weight per panicle	*qPWPP11*	11	Indel_636	21.3	4.4	-0.24	0.23	-
Grain weight per panicle	*qGWPP11*	11	Indel_636	21.3	3.2	-0.21	0.18	-
Grain number per panicle	*qGNPP11*	11	Indel_636	21.3	3.9	-9.62	0.20	-
**Panicle position**	***qPP11***	**11**	**Indel_648**	**25.6**	**3.5**	**-2.05**	**0.11**	**-**

a Physical positions of the closest markers to LOD peaks.

b Additive effect of alleles of MI in each population.

c Percentage of the phenotypic variance explained by each QTL.

### Relationships between spreading habit and yield traits in HOMI-RILs

QTL analysis of yield traits identified QTLs for DTH, PL, PN, PW, SLW, CL, TGW, PWPP, GWPP and GNPP (Table 1), but not for GW or GN. Comparison with previously identified QTLs and genes revealed some candidates: *NAL1* for PN [32, 33], *Ehd1* for DTH [34], *qIN3-6* for CL [35], and *qPL-6* for PL [35].

To determine whether plant spreading habit is related to yield traits in HOMI-RILs, we examined the relationship between indices of spreading habit and QTLs for yield traits. No QTLs for yield traits were detected in the *TAC1* region. We could not find studies of the effect of *TAC1* on traits other than tiller angle. On the other hand, *qSSH6-1* and *qPC1_6* were co-localized with *qCL6*, and the allelic effect on CL differed between HO and MI, suggesting that a longer culm leads to a greater spreading habit. *qPP11* was localized near QTLs for PWPP, GWPP, and GNPP; a possible explanation is that heavier panicles with more grains may spread more. These results suggest that observation of plant architecture using various methods can provide insights into the relationship between plant architecture and yield traits.

## Conclusions

We developed a new method to identify the positions of panicles in individual plants in the field by a commercial camera-UAV. Our method does not need highly specialized facilities or expensive cameras, yet it has high sensitivity for the detection of PP, and is cost-effective, as the red felt and staples cost very little. We detected plant centers in orthomosaic during the vegetative stage to calculate the distance to the flags from the center because it was hard to find the center during the reproductive stage. This technique has been made possible by the development of remote-sensing technology, image analysis, and global positioning system (GPS)-based surveying. The phenotype of PP was similar to but not identical to the phenotypes of LSA, PBA, and SSH; PP is another index of plant architecture in the field. We expect our method to be useful for time-lapse analysis to understand how PP changes over time and is affected by wind, which would provide information on lodging resistance at maturity. PP phenotyping will help in selection during breeding of new cultivars. The integration of genetic analyses with these phenotypes may help to clarify the relationship between plant architecture and crop productivity.

## Supporting information

S1 Fig UAV and the control system used in this study. Phantom 4 Pro (left), the controller with iPad (right) are shown (the grey box behind them is used for their storage).
(TIF)

S2 Fig Orthomosaic of the rice paddy field on 18 June 2018. The orthomosaic was made with 235 digital images taken by our UAV system. Eight points outside the field were used as GCPs to produce the orthomosaic.
(TIF)

S3 Fig Orthomosaic of the rice paddy field on 20 September 2018. The orthomosaic was made with 238 digital images taken by our UAV system. Eight points outside the field were used as GCPs to produce the orthomosaic.
(TIF)

S4 Fig Yield traits of HO and MI RILs. Panicle number (PN), panicle length (PL), and culm length (CL) were assessed from 2 weeks to a month after heading. Panicle weight (PW) was measured after drying. Fertilized grains were isolated from panicles, and grain number (GN) and grain weight (GW) were assessed. Data are means ± standard deviations. *P < 0.05, **P < 0.01 (Student’s *t*-test, *n* = 5).
(TIF)

S5 Fig **Measurement of (A) LSA and (B) PBA with a laser range finder.** Data are means ± standard deviations. Both angles were significantly different between parents (*P < 0.01, Student’s *t*-test, *n* = 5).
(TIF)

S1 Table Meteorological data of the Kannondai District in Tsukuba. Monthly air temperature, relative humidity, sunshine duration, solar radiation, and total precipitation in 2018. The data was extracted from the Weather Data Acquisition System of the Institute for Agro-Environmental Sciences, NARO.
(TIF)

S2 Table Evaluation of color, material, and optimum size of a flag. Bold indicates our final selection. Red was easiest to distinguish from the many senescent yellow leaves and dark green leaves. Narrow polyvinyl chloride (PVC) tape was hard to see as it hung vertically, and hard plastic sheets were difficult to fix to the panicle bases. Soft felt proved easiest to handle and attach. Squares (4.5 cm × 4.5 cm) balanced visibility beneath leaves with not overlapping one another.
(TIF)

S3 Table Principal component analysis of PP, PBA, LSA, and SSH of HOMI-RILs. (TIF)

S1 File Positions and primer sequences of DNA insertion-deletion (indel) markers used for QTL analysis in HOMI-RILs. (XLSX)

S2 File *TAC1* allele sequences of HO and MI. The MI allele of *TAC1* is the same as in Nipponbare. The HO allele of *TAC1* has the single nucleotide polymorphism (SNP) for spreading habit reported in Yu et al. (2007). The position of the mutation is shown in red.
(DOCX)

## References

[1] Yang G, Liu J, Zhao C, Li Z, Huang Y, Yu H, et al Unmanned Aerial Vehicle Remote Sensing for Field-Based Crop Phenotyping: Current Status and Perspectives. Frontiers in plant science. 2017;8:1111 Epub 2017/07/18. 10.3389/fpls.2017.01111 28713402 PMC5492853

[2] Tardieu F, Cabrera-Bosquet L, Pridmore T, Bennett M. Plant Phenomics, From Sensors to Knowledge. Current biology: CB. 2017;27(15):R770–r83. Epub 2017/08/09. 10.1016/j.cub.2017.05.055 . 28787611

[3] Araus JL, Kefauver SC, Zaman-Allah M, Olsen MS, Cairns JE. Translating High-Throughput Phenotyping into Genetic Gain. Trends in plant science. 2018;23(5):451–66. Epub 2018/03/21. 10.1016/j.tplants.2018.02.001 29555431 PMC5931794

[4] Simko I, Jimenez-Berni JA, Sirault XR. Phenomic Approaches and Tools for Phytopathologists. Phytopathology. 2017;107(1):6–17. Epub 2016/09/13. 10.1094/PHYTO-02-16-0082-RVW . 27618193

[5] Gracia-Romero A, Kefauver SC, Fernandez-Gallego JA, Vergara-Diaz O, Nieto-Taladriz MT, Araus JL. UAV and Ground Image-Based Phenotyping: A Proof of Concept with Durum Wheat. Remote Sens. 2019;11(10):25 10.3390/rs11101244 WOS:000480524800102.

[6] Jin XL, Liu SY, Baret F, Hemerle M, Comar A. Estimates of plant density of wheat crops at emergence from very low altitude UAV imagery. Remote Sens Environ. 2017;198:105–14. 10.1016/j.rse.2017.06.007 WOS:000406818500009.

[7] Wang X, Zhang R, Song W, Han L, Liu X, Sun X, et al Dynamic plant height QTL revealed in maize through remote sensing phenotyping using a high-throughput unmanned aerial vehicle (UAV). Scientific reports. 2019;9(1):3458 Epub 2019/03/07. 10.1038/s41598-019-39448-z 30837510 PMC6401315

[8] Weiss M, Baret F. Using 3D Point Clouds Derived from UAV RGB Imagery to Describe Vineyard 3D Macro- Structure. Remote Sens. 2017;9(2):17 10.3390/rs9020111 WOS:000397013700013.

[9] Singh D, Wang X, Kumar U, Gao L, Noor M, Imtiaz M, et al High-Throughput Phenotyping Enabled Genetic Dissection of Crop Lodging in Wheat. Frontiers in plant science. 2019;10:394 Epub 2019/04/26. 10.3389/fpls.2019.00394 31019521 PMC6459080

[10] Tanger P, Klassen S, Mojica JP, Lovell JT, Moyers BT, Baraoidan M, et al Field-based high throughput phenotyping rapidly identifies genomic regions controlling yield components in rice. Scientific reports. 2017;7:42839 Epub 2017/02/22. 10.1038/srep42839 28220807 PMC5318881

[11] Chen Y, Lee WS, Gan H, Peres N, Fraisse C, Zhang YC, et al Strawberry Yield Prediction Based on a Deep Neural Network Using High-Resolution Aerial Orthoimages. Remote Sens. 2019;11(13):21 10.3390/rs11131584 WOS:000477049000076.

[12] Di Gennaro SF, Toscano P, Cinat P, Berton A, Matese A. A Low-Cost and Unsupervised Image Recognition Methodology for Yield Estimation in a Vineyard. Frontiers in plant science. 2019;10:559 Epub 2019/05/28. 10.3389/fpls.2019.00559 31130974 PMC6509744

[13] Duan B, Fang S, Zhu R, Wu X, Wang S, Gong Y, et al Remote Estimation of Rice Yield With Unmanned Aerial Vehicle (UAV) Data and Spectral Mixture Analysis. Frontiers in plant science. 2019;10:204 Epub 2019/03/16. 10.3389/fpls.2019.00204 30873194 PMC6400984

[14] Wang F, Wang F, Zhang Y, Hu J, Huang J, Xie J. Rice Yield Estimation Using Parcel-Level Relative Spectral Variables From UAV-Based Hyperspectral Imagery. Frontiers in plant science. 2019;10:453 Epub 2019/04/27. 10.3389/fpls.2019.00453 31024607 PMC6468049

[15] Gong Y, Duan B, Fang S, Zhu R, Wu X, Ma Y, et al Remote estimation of rapeseed yield with unmanned aerial vehicle (UAV) imaging and spectral mixture analysis. Plant methods. 2018;14:70 Epub 2018/08/29. 10.1186/s13007-018-0338-z 30151031 PMC6102863

[16] Peng J, Richards DE, Hartley NM, Murphy GP, Devos KM, Flintham JE, et al 'Green revolution' genes encode mutant gibberellin response modulators. Nature. 1999;400(6741):256–61. Epub 1999/07/27. 10.1038/22307 . 10421366

[17] Sasaki A, Ashikari M, Ueguchi-Tanaka M, Itoh H, Nishimura A, Swapan D, et al Green revolution: a mutant gibberellin-synthesis gene in rice. Nature. 2002;416(6882):701–2. Epub 2002/04/19. 10.1038/416701a . 11961544

[18] Spielmeyer W, Ellis MH, Chandler PM. Semidwarf (sd-1), "green revolution" rice, contains a defective gibberellin 20-oxidase gene. Proceedings of the National Academy of Sciences of the United States of America. 2002;99(13):9043–8. Epub 2002/06/22. 10.1073/pnas.132266399 12077303 PMC124420

[19] Wang B, Smith SM, Li J. Genetic Regulation of Shoot Architecture. Annual review of plant biology. 2018;69:437–68. Epub 2018/03/20. 10.1146/annurev-arplant-042817-040422 . 29553800

[20] Mathan J, Bhattacharya J, Ranjan A. Enhancing crop yield by optimizing plant developmental features. Development (Cambridge, England). 2016;143(18):3283–94. Epub 2016/09/15. 10.1242/dev.134072 . 27624833

[21] Teichmann T, Muhr M. Shaping plant architecture. Frontiers in plant science. 2015;6:233 Epub 2015/04/29. 10.3389/fpls.2015.00233 25914710 PMC4390985

[22] Sakamoto T, Morinaka Y, Ohnishi T, Sunohara H, Fujioka S, Ueguchi-Tanaka M, et al Erect leaves caused by brassinosteroid deficiency increase biomass production and grain yield in rice. Nature biotechnology. 2006;24(1):105–9. Epub 2005/12/22. 10.1038/nbt1173 . 16369540

[23] Wang S, Wu K, Qian Q, Liu Q, Li Q, Pan Y, et al Non-canonical regulation of SPL transcription factors by a human OTUB1-like deubiquitinase defines a new plant type rice associated with higher grain yield. Cell research. 2017;27(9):1142–56. Epub 2017/08/05. 10.1038/cr.2017.98 28776570 PMC5587855

[24] Atkin OK, Loveys BR, Atkinson LJ, Pons TL. Phenotypic plasticity and growth temperature: understanding interspecific variability. Journal of experimental botany. 2006;57(2):267–81. Epub 2005/12/24. 10.1093/jxb/erj029 . 16371402

[25] Jagadish SV, Murty MV, Quick WP. Rice responses to rising temperatures—challenges, perspectives and future directions. Plant, cell & environment. 2015;38(9):1686–98. Epub 2014/08/22. 10.1111/pce.12430 . 25142172

[26] Blackman BK. Changing Responses to Changing Seasons: Natural Variation in the Plasticity of Flowering Time. Plant physiology. 2017;173(1):16–26. Epub 2016/11/23. 10.1104/pp.16.01683 27872243 PMC5210766

[27] Ogawa D, Yamamoto E, Ohtani T, Kanno N, Tsunematsu H, Nonoue Y, et al Haplotype-based allele mining in the Japan-MAGIC rice population. Scientific reports. 2018;8(1):4379 Epub 2018/03/14. 10.1038/s41598-018-22657-3 29531264 PMC5847589

[28] Weather Data Acquisition System of Institute for Agro-Environmental Sciences N. http://www.naro.affrc.go.jp/org/niaes/aws/ (Japanese language version).

[29] Lander ES, Green P, Abrahamson J, Barlow A, Daly MJ, Lincoln SE, et al MAPMAKER: an interactive computer package for constructing primary genetic linkage maps of experimental and natural populations. Genomics. 1987;1(2):174–81. Epub 1987/10/01. 10.1016/0888-7543(87)90010-3 . 3692487

[30] Yonemaru J, Mizobuchi R, Kato H, Yamamoto T, Yamamoto E, Matsubara K, et al Genomic regions involved in yield potential detected by genome-wide association analysis in Japanese high-yielding rice cultivars. BMC genomics. 2014;15:346 Epub 2014/06/03. 10.1186/1471-2164-15-346 24885019 PMC4035073

[31] Yu B, Lin Z, Li H, Li X, Li J, Wang Y, et al TAC1, a major quantitative trait locus controlling tiller angle in rice. The Plant journal: for cell and molecular biology. 2007;52(5):891–8. Epub 2007/10/03. 10.1111/j.1365-313X.2007.03284.x . 17908158

[32] Fujita D, Trijatmiko KR, Tagle AG, Sapasap MV, Koide Y, Sasaki K, et al NAL1 allele from a rice landrace greatly increases yield in modern indica cultivars. Proceedings of the National Academy of Sciences of the United States of America. 2013;110(51):20431–6. Epub 2013/12/04. 10.1073/pnas.1310790110 24297875 PMC3870739

[33] Yano K, Yamamoto E, Aya K, Takeuchi H, Lo PC, Hu L, et al Genome-wide association study using whole-genome sequencing rapidly identifies new genes influencing agronomic traits in rice. Nature genetics. 2016;48(8):927–34. Epub 2016/06/21. 10.1038/ng.3596 . 27322545

[34] Doi K, Izawa T, Fuse T, Yamanouchi U, Kubo T, Shimatani Z, et al Ehd1, a B-type response regulator in rice, confers short-day promotion of flowering and controls FT-like gene expression independently of Hd1. Genes & development. 2004;18(8):926–36. Epub 2004/04/14. 10.1101/gad.1189604 15078816 PMC395851

[35] Yamamoto T, Taguchi-Shiobara F, Ukai Y, Sasaki T, Yano M. Mapping quantitative trait loci for days-to-heading, and culm, panicle and internode lengths in a BC1F3 population using an elite rice variety, Koshihikari, as the recurrent parent. Breeding science. 2001;51(2):63–71. 10.1270/jsbbs.51.63 WOS:000170277000001.

