# Supplementary figures and images for: Surveillance of panicle positions by unmanned aerial vehicle to reveal morphological features of rice
Source: PLoS One. 2019 Oct 31;14(10):e0224386. doi: 10.1371/journal.pone.0224386 (PMC6822732; doi:10.1371/journal.pone.0224386)

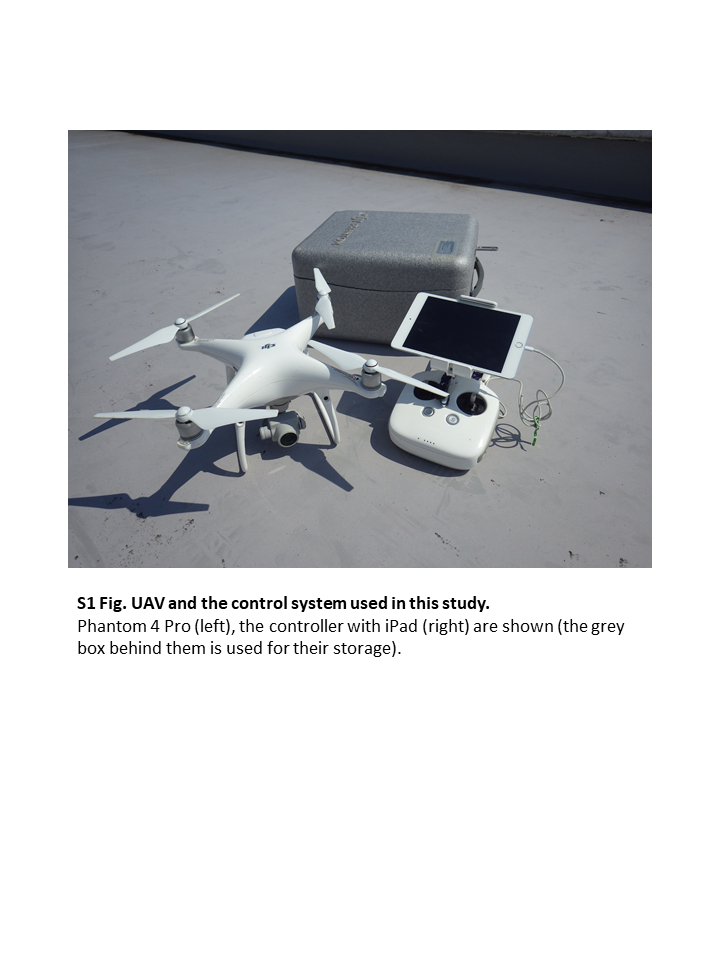

Supplement: S1 Fig — Phantom 4 Pro (left), the controller with iPad (right) are shown (the grey box behind them is used for their storage). (TIF) [file pone.0224386.s001.TIF]

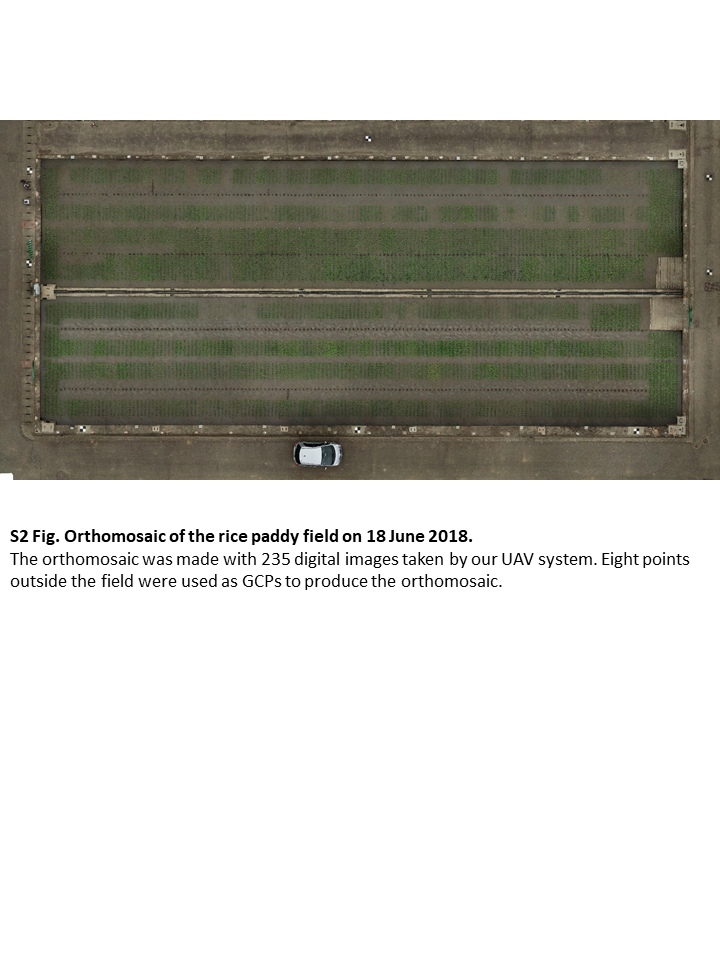

Supplement: S2 Fig — The orthomosaic was made with 235 digital images taken by our UAV system. Eight points outside the field were used as GCPs to produce the orthomosaic. (TIF) [file pone.0224386.s002.TIF]

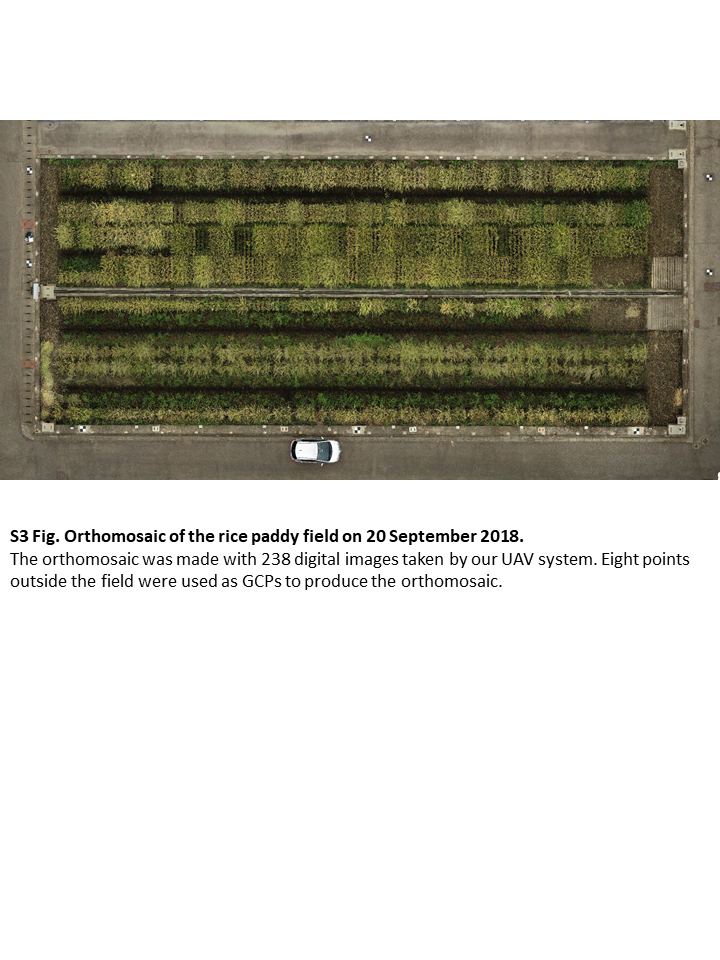

Supplement: S3 Fig — The orthomosaic was made with 238 digital images taken by our UAV system. Eight points outside the field were used as GCPs to produce the orthomosaic. (TIF) [file pone.0224386.s003.TIF]

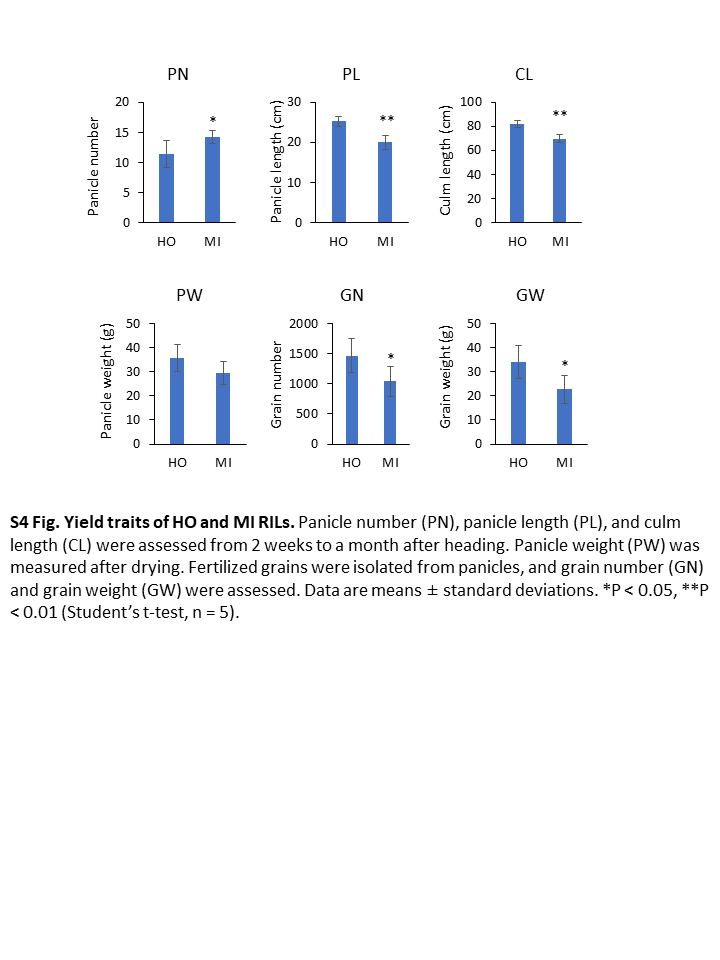

Supplement: S4 Fig — Panicle number (PN), panicle length (PL), and culm length (CL) were assessed from 2 weeks to a month after heading. Panicle weight (PW) was measured after drying. Fertilized grains were isolated from panicles, and grain number (GN) and grain weight (GW) were assessed. Data are means ± standard deviations. *P < 0.05, **P < 0.01 (Student’s t-test, n = 5). (TIF) [file pone.0224386.s004.TIF]

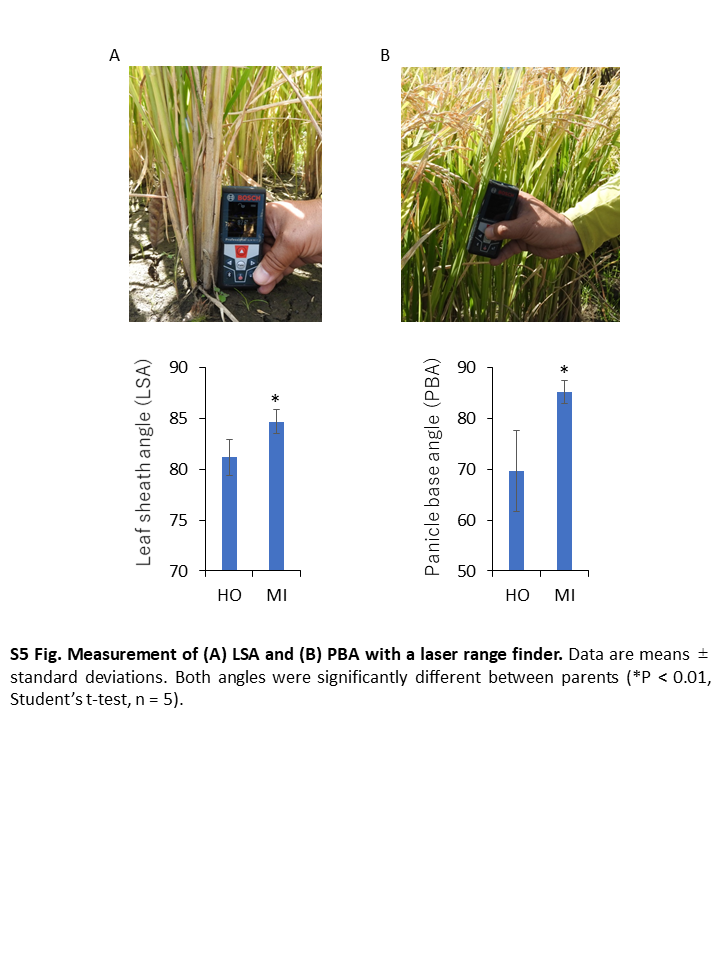

Supplement: S5 Fig — Measurement of (A) LSA and (B) PBA with a laser range finder. Data are means ± standard deviations. Both angles were significantly different between parents (*P < 0.01, Student’s t-test, n = 5). (TIF) [file pone.0224386.s005.TIF]

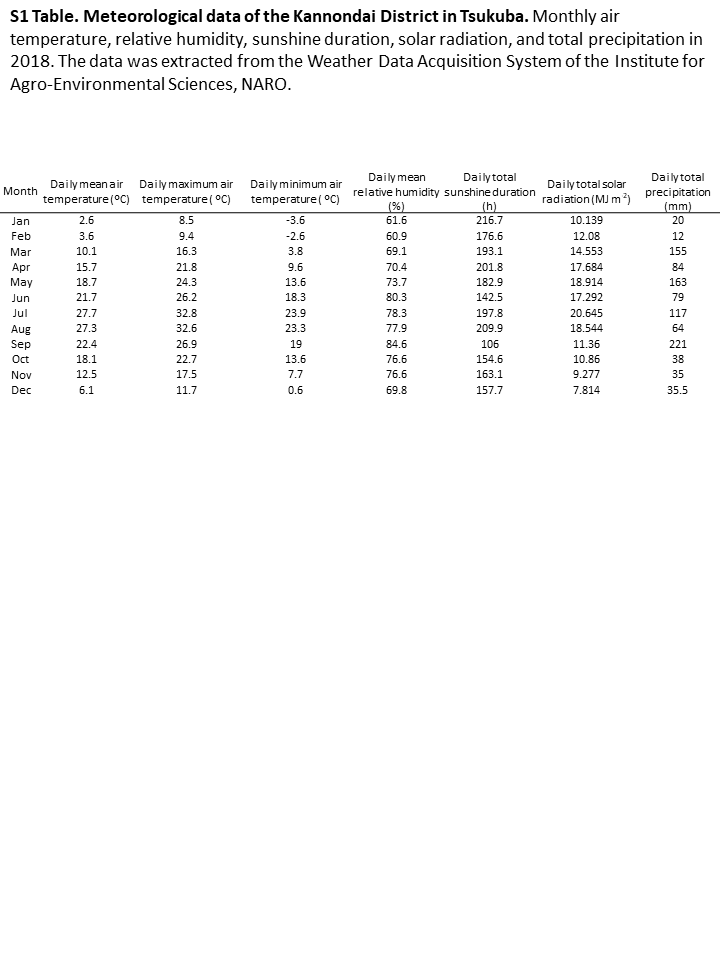

Supplement: S1 Table — Monthly air temperature, relative humidity, sunshine duration, solar radiation, and total precipitation in 2018. The data was extracted from the Weather Data Acquisition System of the Institute for Agro-Environmental Sciences, NARO. (TIF) [file pone.0224386.s006.TIF]

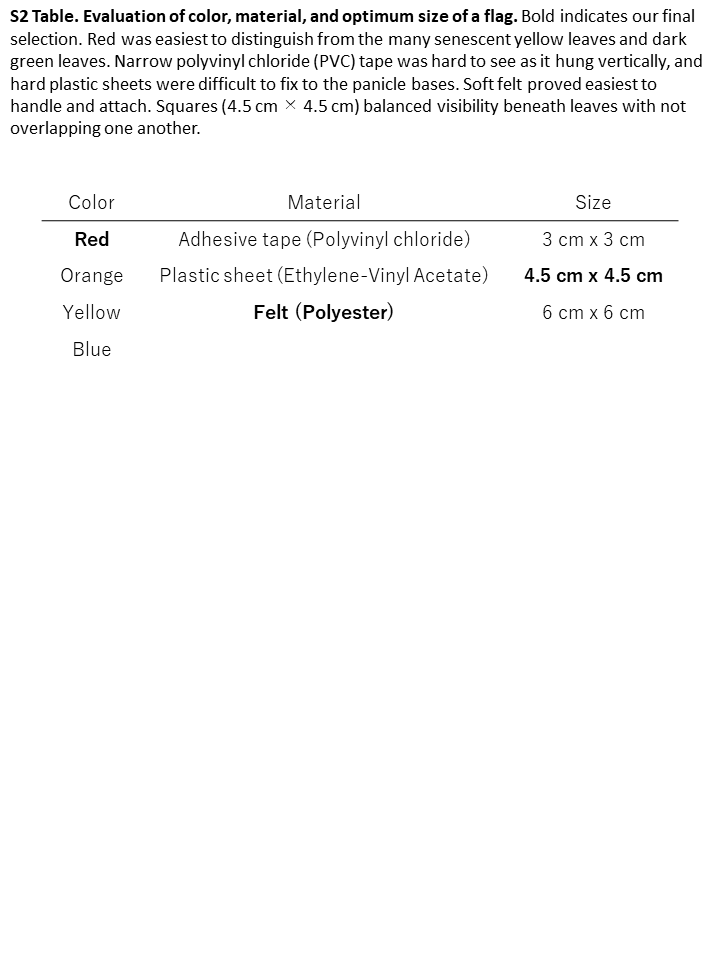

Supplement: S2 Table — Bold indicates our final selection. Red was easiest to distinguish from the many senescent yellow leaves and dark green leaves. Narrow polyvinyl chloride (PVC) tape was hard to see as it hung vertically, and hard plastic sheets were difficult to fix to the panicle bases. Soft felt proved easiest to handle and attach. Squares (4.5 cm × 4.5 cm) balanced visibility beneath leaves with not overlapping one another. (TIF) [file pone.0224386.s007.TIF]

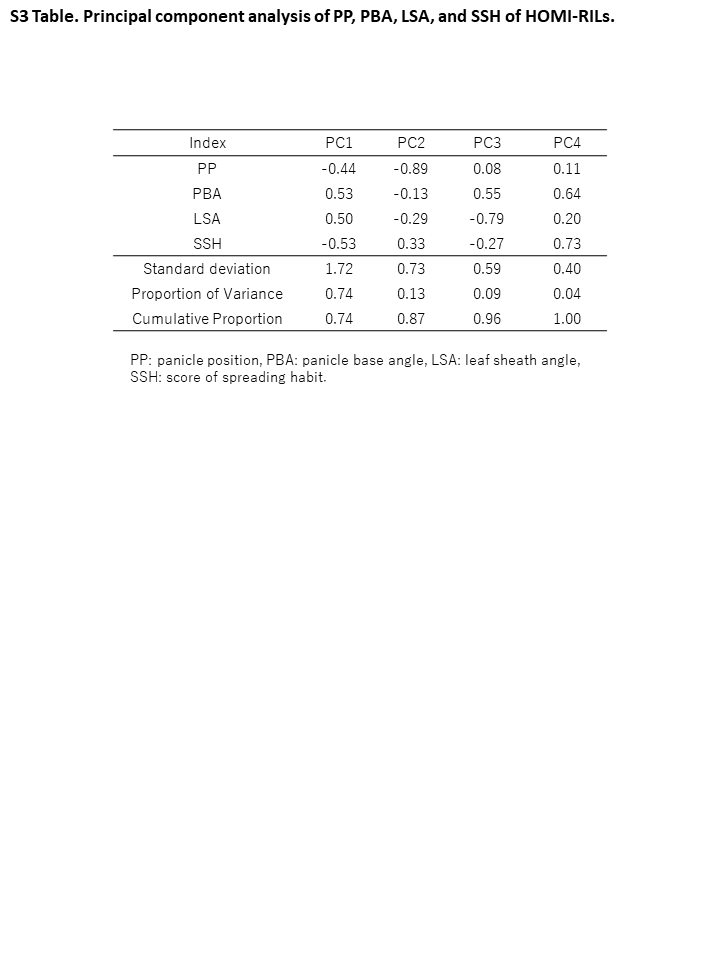

Supplement: S3 Table — (TIF) [file pone.0224386.s008.TIF]
