# Supplementary material for: Surveillance of panicle positions by unmanned aerial vehicle to reveal morphological features of rice
Source: PLoS One. 2019 Oct 31;14(10):e0224386. doi: 10.1371/journal.pone.0224386 (PMC6822732; doi:10.1371/journal.pone.0224386)
Supplement: S2 File — The MI allele of TAC1 is the same as in Nipponbare. The HO allele of TAC1 has the single nucleotide polymorphism (SNP) for spreading habit reported in Yu et al. (2007). The position of the mutation is shown in red. (DOCX) [file pone.0224386.s010.docx]

**S2 File. TAC1 allele sequences of HO and MI.** The MI allele of TAC1 is the same as in Nipponbare. The HO allele of TAC1 has the single nucleotide polymorphism (SNP) for spreading habit reported in Yu et al. (2007). The position of the mutation is shown in red.

>TAC1_MI

GAGCTACTGTCTGGCTTTCTCTTCTGGTTTCATATTGGTTCTAGAGAGATGGCTCTAAAGGTACACAGTTAGTTCATGCTGATACACCATTTGTCCGTGTATAGTGTATGCTGTAAAGTACAGGTCTTTGCAGTTTCTGTCTCTCTTAAACAAGTGCGTTGGATTCAACTCACTGTTCTTATATCTAATGAGATTACTGGTGATTTATGTTTTTATGGCACTACCAGGTGTTCAATTGGCTGAATCGGAAGAAGCATTCTAATGTCGAGTATTGCACCATCAATGAGAACAAGGGTAAGGATACCAGGATATCTCTCTTTTATGTACCACATGTTTTGTTGTTCTCATGTTGTCAACTCTTGTTTCTTCTCTTCTATTTTTTTTCCTGATGGAGTAGCCATGGAAGAGAAGGAAGACTCTCTGCGTGCAAGTGTGACTGAGCAAGACACTGAGGCCCTGCTGCTCCGTGATGTGCTTATTAATGGTATACTTGCAATTGGCACGCTGGGCCACAATGTAAACTCACTCTGTCCTGAGTCCTGTATTGAACAAGATGAGCCCATCATCATGTGTGATGAGAAAGTGGAACAAGAGAAGTGCGAAGAAGAAAAGGCTGAGGCTAAACAGGACACACCAGTTACAGCACCAAGTGAACCGGCATCTGCTCTTGAGCCTGCCAAGATGCACTCATCATCGATGAAAGAAGACAACTTCATGTGCTTTGTGAAGGAGGAAATCCTAATGCATGGCATGGAAGTGGAAGATGTTCCTAACATCCAGGAACGACCACTTCTGATGTTAGAGAAGGTGGAGAAAGTGAGAACTACACTTGCCGATCTATTTGCTGCAGAAGCATTCTCATCAAGTGATGCAGAGGATAAGTGTTACCCGAAAATCGTCATTGTTGCTGGGGCATCCACTTCAAAGCCTACGTCGTGCATGGAGAAGATGCATCACAAGAAGCCAACAAAACCAACGTCAAAGCCGCTGAAGGCTACAAGAAAATTAAGTCGAGTATGGTTTTCTTCGTCTCTGCTTTATTTGTTAAGCCTTGTTACATTATATTTTACTAGTCTGGAATTTATTACACTTTGTTCCATGGTCAGTGCGCCACATCTTACACATATGTTTTACCATATAGCTACAACTGGAGTAAGCATTATGCATCTAGCCAGTCTTTAGTGGTAAATGATCACTGACCTGGAATGCACCTTTTTGGTTTTTGTCATCTGTAAAATAAGTAGGTCATGAGGAAGATGTTGGGGAAGAAGATCCACCCAGAGCAGCTCAATGGACGTAGCAATGCAGAGGGCCCTGTCACTGCATAATGCTAGGTTTGTGGACAAAAGTTTCCTTTCTCTATCTGGCAATTTATTACCTAGAGTTTTTTTAAAGCTGTCTGTTGAACTATACAGGGCTCAAGGCCCTTGTGTTTTGCATACGATTTTGTACCTTTCCAAATTTTCTTGATTTTTACTTGGATTCCATTGTTTGTAATAAAATTAGGTTAATATCTGAGGTAGTATTTGCATAGAATGGATACCTTCTACCAAAGTTATATTTGTTTGTGGGTCCTAGAGCTAGCATGACCCACGAATCACATTCAAGAATATAGTAATTCGTTTAGGCTACATTTCACTAGGAATAGAACATATGAATATTCTCAGGTTTAATAAGGCAGATGCAAAAAAGAACTGAATAGCACAGGAAAGTAATTTTTCCATTTCAAGCTTCTCACAGTCAAAAACAAGTAATGGTTCAAAAATTGAATATTCTATCACCTGTTGCCTCCATTTATGTGGAACTCACAAGAGGGTCTAAGTGCTGCTTGACACTCTGACCTGTATTTAAATAAAATGTTATCACCTATTGCCTCTGCTTATTATTGGGATCTCAAAACACAATCTATTACTGTGTATGCCTTGGCATTTTTTAAAATCTGAGCTGCCCCCTTATTCTTCACATTTTCTCAGAAAACCATATAAACTTTTAGATAAATGAAGCTTTTATTGATCTCAGACAATTACATCAAGTTGATAGAACCAAACTAAGAACACTTCTGGCCTCTGATAATGGAACTGCTGTTTGTTTAAGTAGAAAGAATGGTGTGTGTATTTTAACGGTTGGTGATTGGGAATTGGGATGCAGTGACCATGGAGACTGTGTTCTTCAGAATATTTTTAAGAAATAGTGTTTTCGGTGGATCCTATCATGGAACTGCCTATGGTGGAAAGAGCAATACGAGTTGAAACATATAGGGATTAGGAAGCATGGGTTCGGACTAAAATGCATTCGTTCCTGCCATGATGCAAAGCTTCATTCGAAGCAAAATGAGCACATGCCATGGTACAGTGGCGTGCAACGAGCACTGCAATTGCACACGACGTGCATGTTCTGAAAAAGGCCAGTGGACACTAGACATGCAGCTAATCCTCTTTTGATGAATTCTATCATGAAACCAATGGATTCGTTGAAAATCTGAACAAATTGATGGATTTGGTCCCAGCTGTAGAAAGGTTGGCAAACTCTTCTCGCTCCTGTATTATTGTAGGCTGGAGCTATGATGGACCAAATCATCAGAAGCAAATTTCACCATATATAAGCATCTACTTTTATCTTTTTCTCTATTTTAAAATGTGATCATAGTGAAGTTATATACATTCTATCTGCTTAACGCTCCACATTATCCAAAAAAATGCATCACACTCCATATTGGGAACACGTTGGTGGTTCTGAACCAAAGTCGATCTCTTGATGCCAATTTTTTTTTTGAGCTGGTTGAGTAGTCGAACTGGGAACAATTACTGCTTGAGGCTACAAAATTTCTGGCCGACAATACGTCTTGATCAGGAACTGACTAAAGAAATTCCCTTTCACCTTTTGCGGGATTTGTTGGATCCATCCTCACGCTTCGGATTCCTTGCTCAAGAGAAACATCCATGCATATCGTCGACAGCGTTCGTTCAGTCCTCTTCCTTTTGTTGTTGGTGCTGTTGTTATTATTGTTATTGTTATTGCTGCCTATTCGCTCGCCAGTGATGAAGAATAGTCCTGCCTATATTTGCCTGTAGTACATTGTAAAGCTACAGTTGACGTGTCTTGTAAGACCCTTATTATTATTGTCCATACCACGACGTCTC

>TAC1_HO

GAGCTACTGTCTGGCTTTCTCTTCTGGTTTCATATTGGTTCTAGAGAGATGGCTCTAAAGGTACACAGTTAGTTCATGCTGATACACCATTTGTCCGTGTATAGTGTATGCTGTAAAGTACAGGTCTTTGCAGTTTCTGTCTCTCTTAAACAAGTGCGTTGGATTCAACTCACTGTTCTTATATCTAATGAGATTACTGGTGATTTATGTTTTTATGGCACTACCAGGTGTTCAATTGGCTGAATCGGAAGAAGCATTCTAATGTCGAGTATTGCACCATCAATGAGAACAAGGGTAAGGATACCAGGATATCTCTCTTTTATGTACCACATGTTTTGTTGTTCTCATGTTGTCAACTCTTGTTTCTTCTCTTCTATTTTTTTCCCTGATGGAGTAGCCATGGAAGAGAAGGAAGACTCTCTGCGTGCAAGTGTGACTGAGCAAGACACTGAGGCCCTGCTGCTCCGTGATGTGCTTATTAATGGTATACTTGCAATTGGCACGCTGGGCCACAATGTAAACTCACTCTGTCCTGAGTCCTGTATTGAACAAGATGAGCCCATCATCATGTGTGATGAGAAAGTGGAACAAGAGAAGTGCGAAGAAGAAAAGGCTGAGGCTAAACAGGACACACCAGTTACAGCACCAAGTGAACCGGCATCTGCTCTTGAGCCTGCCAAGATGCACTCATCATCGATGAAAGAAGACAACTTCATGTGCTTTGTGAAGGAGGAAATCCTAATGCATGGCATGGAAGTGGAAGATGTTCCTAACATCCAGGAACGACCACTTCTGATGTTAGAGAAGGTGGAGAAAGTGAGAACTACACTTGCCGATCTATTTGCTGCAGAAGCATTCTCATCAAGTGATGCAGAGGATAAGTGTTACCCGAAAATCGTCATTGTTGCTGGGGCATCCACTTCAAAGCCTACGTCGTGCATGGAGAAGATGCATCACAAGAAGCCAACAAAACCAACGTCAAAGCCGCTGAAGGCTACGAGAAAATTAAGTCGAGTATGGTTTTCTTCGTCTCTGCTTTATTTGTTAAGCCTTGTTACATTATATTTTACTAGTCTGGAATTTATAACACTTTGTTCCATGGTCAGTGCGCCACATCTTACACATATGTTTTACCATATAGCTACAACTGGAGTAAGCATTATGCATCTAGCCAGTCTTTAGTGGTAAATGATCACTGACCTGGAATGCACCTTTTTGGTTTTTGTCATCTGTAAAATAAGTAGGTCATGAGGAAGATGTTGGGGAAGAAGATCCACCCAGAGCAGCTCAATGGACGTAGCAATGCAGAGGGCCCTGTCACTGCATAATGCTAGGTTTGTGGACAAAAGTTTCCTTTCTCTATCTGGCAATTTATTACCTAGAGTTTTTTTAAAGCTGTCTGTTGAACTATGCAGGGCTCAAGGCCCTTGTGTTTTGCATACGATTTTGTACCTTTCCAAATTTTCTTGATTTTTACTTGGATTCCATTGTTTGTAATAAAATTAGGTTAATATCTGAGGTAGTATTTGCATAGAATGGATACCTTCTACCAAAGTTATATTTGTTTGTGGGTCCTAGAGCTAGCATGACCCACGAATCACATTCAAGAATATAGTAATTCGTTTAGGCTACATTTCACTAGGAATAGAACATATGAATATTCTCAGGTTTAATAAGGCAGATGCAAAAAAGAACTGAATAGCACAGGAAAGTAATTTTTCCATTTCAAGCTTCTCACAGTCAAAAACAAGTAATGGTTCAAAAATTGAATATTCTATCACCTGTTGCCTCCATTTATGTGGAACTCACAAGAGGGTCTAAGTGCTGCTTGACACTCTGACCTGTATTTAAATAAAATGTTATCACCTATTGCCTCTGCTTATTATTGGGATCTCAAAACACAATCTATTACTGTGTATGCCTTGGCATTTTTTAAAATCTGAGCTGCCCCCTTATTCTTCACATTTTCTCAGAAAACCATATAAACTTTTAGATAAATGAAGCTTTTATTGATCTCAGACAATTACATCAAGTTGATAGAACCAAACTAAGAACACTTCTGGCCTCTGATAATGGAACTGCCGTTTGTTTAAGTAGAAAGAATGGTGTGTGTATTTTAACGGTTGGTGATTGGGAATTGGGATGCAGTGACCATGGAGACTGTGTTCTAGTGTTTTCGGTGGATCCTATCATGGAACTGCCTATGGTGGAAAGAGCAATACGAGTTGAAACATATAGGGATTAGGAAGCATGGGTTCGGACTAAAATGCATTCGTTCCTGCCATGATGCAAAGCTTCATTCGAAGCAAAATGAGCACATGCCATGGTACAGTGGCGTGCAACGAGCACTGCAATTGCACACGATGTGCATGTTCTGAAAAAGGCCAGTGGACACTAGACATGCAGCTAATCCTCTTTTGATGAATTCTATCATGAAACCAATGGATTCGTTGAAAATCTGAACAAATTGATGGATTTGGTCCCAGCTGTAGAAAGGTTGGCAAACTCTTCTCGCTCCTGTATTATTGTAGGCTGGAGCTATGATGGACCAAATCATCAGAAGCAAATTTCACCATATATAAGCATCTACTTTTATCTTTTTCTCTATTTTAAAATGTGATCATAGTGAAGTTATGTACATTCTATCTGCTTAACGCTCCACATTATCTAAAAAAATGCATCACACTCCATATTGGGAACACGTTGGTGGTTCTGAACCAAAGTCGATCTCTTGATGCCAATTTTTTTTTGAGCTGGTTGAGTAGTCGAACTGGGAACAATTACTGCTTGAGTCTACAAAATTTCTGGCCGACAATACGTCTTGATCAGGAACTGACTAAAGAAATTCCCTTTCACCTTTTGCAGGATTTGTTGGATCCATCCTCACGCTTCGGATTCCTTGCTCAAGAGAAACATCCATGCATATCGTCGACAGCGTTCGTTCAGTCCTCTTCCTTTTGTTGTTGTTGCTGTTGTTATTATTGTTATTGTTATTGTTATTGCTGCCTATTCGCTCGCCAGTGATGAAGAATAGTCCTGCCTATATTTGCCTGTAGTACATTGTAAAGCTACAGTTGACGTGTCTTGTAAGACCCTTATTATTATTGTCCATACCACGACGTCTC
